# Life history strategies of stream fishes linked to predictors of hydrologic stability

**DOI:** 10.1002/ece3.8861

**Published:** 2022-04-29

**Authors:** Nathaniel P. Hitt, Andrew P. Landsman, Richard L. Raesly

**Affiliations:** ^1^ U.S. Geological Survey U.S. Department of the Interior Eastern Ecological Science Center Kearneysville West Virginia USA; ^2^ National Park Service U.S. Department of the Interior Chesapeake and Ohio Canal National Historical Park Williamsport Maryland USA; ^3^ 3445 Department of Biology Frostburg State University Frostburg Maryland USA

**Keywords:** freshwater fish, hydrology, karst, life history, streams

## Abstract

Life history theory provides a framework to understand environmental change based on species strategies for survival and reproduction under stable, cyclical, or stochastic environmental conditions. We evaluated environmental predictors of fish life history strategies in 20 streams intersecting a national park within the Potomac River basin in eastern North America. We sampled stream sites during 2018–2019 and collected 3801 individuals representing 51 species within 10 taxonomic families. We quantified life history strategies for species from their coordinates in an ordination space defined by trade‐offs in spawning season duration, fecundity, and parental care characteristic of opportunistic, periodic, and equilibrium strategies. Our analysis revealed important environmental predictors: Abundance of opportunistic strategists increased with low‐permeability soils that produce flashy runoff dynamics and decreased with karst terrain (carbonate bedrock) where groundwater inputs stabilize stream flow and temperature. Conversely, abundance of equilibrium strategists increased in karst terrain indicating a response to more stable environmental conditions. Our study indicated that fish community responses to groundwater and runoff processes may be explained by species traits for survival and reproduction. Our findings also suggest the utility of life history theory for understanding ecological responses to destabilized environmental conditions under global climate change.

## Graphical abstract

We show that species traits for survival and reproduction explain fish community responses to groundwater and runoff processes in headwater streams. Our results also indicate the utility of life history theory for understanding ecological responses to destabilized environmental conditions under global climate change.

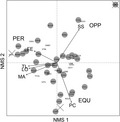

## 1 INTRODUCTION

Species face trade‐offs between energetic investments in growth, reproduction, and survival that shape the evolution of life history strategies under different environmental conditions (Gadgil & Bossert, 1970; Levins, 1967; Southwood, 1977; Stearns, 1992). Stable or predictable environmental conditions can benefit long‐lived species with delayed maturation and low fecundity (i.e., *K*‐selected species), whereas unpredictable environmental conditions can benefit short‐lived species with rapid maturation and low juvenile survivorship (i.e., *r*‐selected species; Pianka, 1970). Environmental stability also shapes the evolution of iteroparous and semelparous reproduction in many taxonomic groups (Cole, 1954; Murphy, 1968) as well as the distribution and abundance of annual and perennial plants (Schaffer, 1974). Life history theory provides a fundamental framework to understand environmental change based on species strategies for survival and reproduction under stable, cyclical, or stochastic environmental conditions.

Fishes constitute an important model for life history research because they occupy diverse environmental conditions and have been undergoing natural selection since the Cambrian Period, longer than any other vertebrate group (Long et al., 2019). Freshwater and marine fishes exhibit life history strategies within a trilateral continuum defined by opportunistic, periodic, and equilibrium endpoints (Heino et al., 2013; Mims et al., 2010; Winemiller, 1992; Winemiller & Rose, 1992). Opportunistic strategies are demonstrated by short‐lived, small‐bodied species with early maturation, and low juvenile survivorship rates. Species that exhibit periodic strategies invest in growth and fecundity through delayed reproduction, whereas species exhibiting equilibrium strategies exhibit low fecundity but compensate for this with increased juvenile survivorship through parental care. Although some fish species exemplify a single life history strategy, most exhibit intermediate strategies between opportunistic, periodic, and equilibrium endpoints (Hitt et al., 2020; King & McFarlane, 2003; Winemiller & Rose, 1992). For example, fish species in the genus *Etheostoma* (Percidae) can exhibit early maturation as well as parental care (Frimpong & Angermeier, 2009; Winemiller & Rose, 1992), thus combining attributes of opportunistic and equilibrium strategies.

Life history theory has utility for understanding hydrologic controls on freshwater fish populations and communities. In lotic environments, flow regulation from dams and reservoirs can increase abundance of equilibrium strategist fishes (Kominoski et al., 2018; McManamay & Frimpong, 2015; Mims & Olden, 2013; Olden et al., 2006; Perkin et al., 2017), whereas hydrologic spates and droughts can increase abundance of opportunistic strategists (Hitt et al., 2020; Magoulick et al., 2021; Malone et al., 2021; Mims & Olden, 2013; Olden & Kennard, 2010). Strong seasonal fluctuations in flow, such as seasonal inundation of floodplains, are associated with periodic strategist fishes (Tedesco et al., 2008). Spatial patterns also indicate the importance of hydrologic controls on fish life history diversity: Species found in flashy headwater streams tend to have smaller bodies, shorter lifespans, and earlier maturation than species found in more stable conditions downstream (Schlosser, 1990). Similarly, large‐bodied, long‐lived fishes are more abundant where pool habitats are common and less abundant where turbulent riffle habitats are prevalent (Lamouroux et al., 2002).

Life history theory can also inform an ecological understanding of land use and climate change. Extreme precipitation events have increased over recent decades (Easterling et al., 2000; Gershunov et al., 2019), and many river systems show increasing flow variation in response (Coumou & Rahmstorf, 2012; Milly et al., 2008; Rahmstorf & Coumou, 2011; Ward et al., 2015). Urbanization can also increase flashiness and decrease stability of downstream flows (Anderson, 1970; O'Driscoll et al., 2010; Sauer et al., 1983), and therefore, cumulative effects of land use and climate change are expected to increase flow variation and decrease predictability of annual flow regimes (Miller & Hutchins, 2017; Zhou et al., 2016). However, groundwater inputs can moderate stream flow and temperature fluctuations (Kaandorp et al., 2019; Meisner et al., 1988; Poff & Ward, 1989; Snyder et al., 2015), and these effects may be particularly important in karst terrain where extensive aquifers permeate weathered carbonate bedrock materials (Bonacci et al., 2009). For instance, fish community composition can transition sharply where karst groundwater enters a stream (Coulter & Galarowicz, 2015), and temporal stability of stream fish communities has been attributed to the stabilizing effects of karstic groundwater inputs (Kollaus et al., 2015; Magoulick et al., 2021).

In this study, we applied life history theory to evaluate the role of hydrologic stability on stream fish community composition in the Potomac River basin of eastern North America. We tested our expectations that anthropogenic land use and flashy stream flows benefit species with opportunistic life history strategies rather than equilibrium or periodic strategies because species with rapid development and extended spawning seasons can rebound quickly from environmental disturbances. We also hypothesized that groundwater inputs increase equilibrium life history strategies due to stabilized hydrologic conditions that benefit investment in parental care for juvenile survival.

## 2 MATERIAL AND METHODS

### 2.1 Study area and sampling design

**FIGURE 1:**
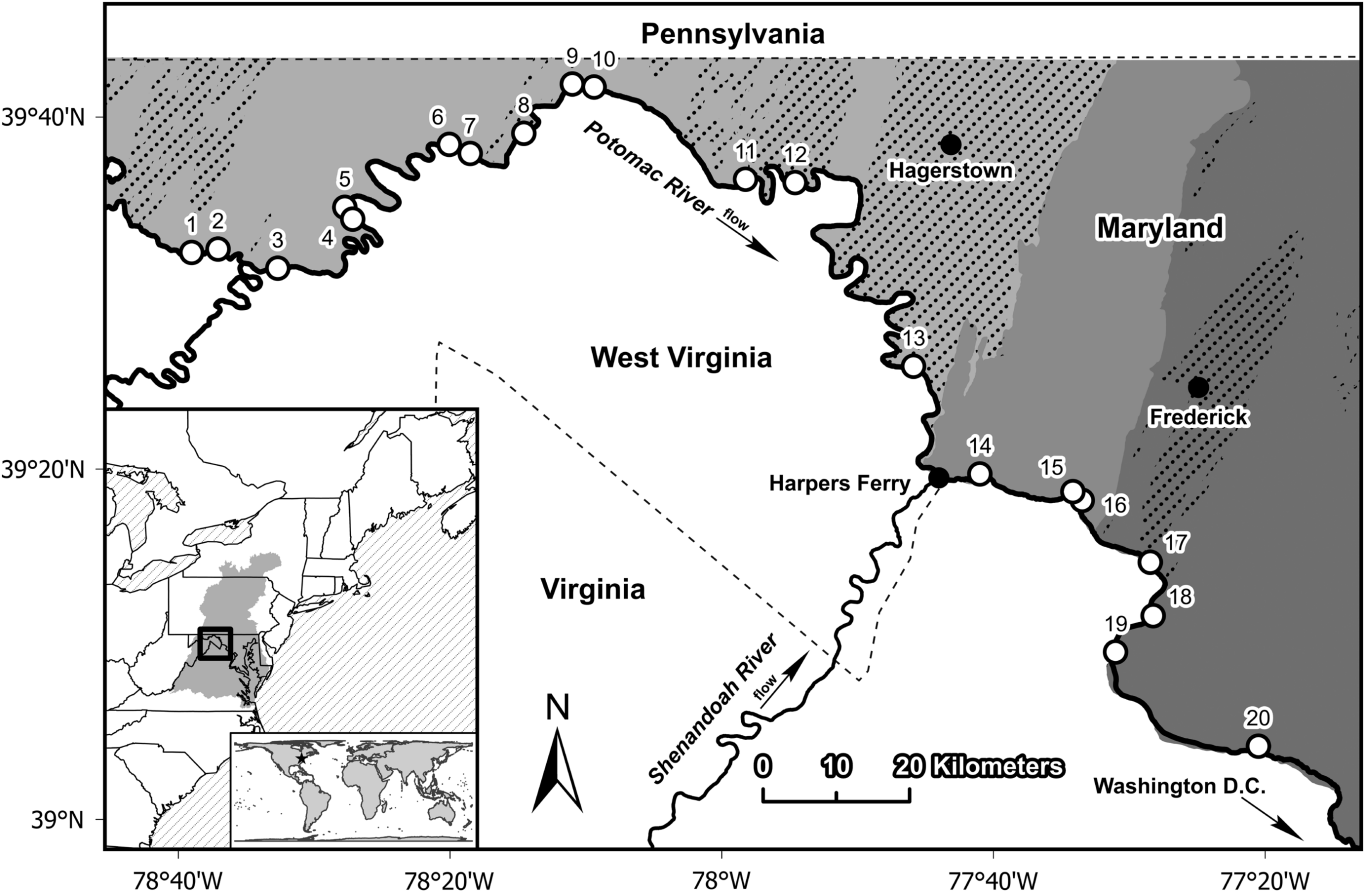
Study area within the Potomac River basin of eastern North America. Open circles show sample site locations (Table A1 in Appendix 1) and site codes (Table 1). Sites were located on streams within the Chesapeake and Ohio Canal National Historical Park near the Potomac River. Shaded areas show physiographic regions within Maryland from west to east as the Ridge and Valley, Blue Ridge, and Piedmont (Reger & Cleaves, 2008), and the stippled areas show regions of karst geology (Weary & Doctor, 2014). The shaded region in the inset map shows the Chesapeake Bay watershed

Our study area encompassed streams intersecting the Chesapeake and Ohio Canal National Historical Park (C&O Canal), an administrative unit of the U.S. National Park Service located in the headwaters of the Chesapeake Bay in eastern North America (Figure 1). A primary management objective of the National Park Service and the C&O Canal is to support biological conservation and diverse natural ecosystems (NPS, 2006). The C&O Canal extends for nearly 300 km along the north bank of the Potomac River and is characterized as a narrow band of forest within watersheds of mixed forest, agricultural, and urban land cover. The study area extends through three physiographic regions (Ridge and Valley, Blue Ridge, and Piedmont) and includes areas of karst geology within the Ridge and Valley province (Doctor et al., 2014; Weary & Doctor, 2014). Karst groundwater flow paths in this region exhibit spatially and temporally complex patterns typically associated with faults and fractured rock layers (Evaldi et al., 2009; Kozar et al., 1991) rather than conduit‐type flow paths characteristic of cave systems.

We selected 20 streams from across the length of the C&O Canal that represented each physiographic region (Figure 1; Table A1 in Appendix 1). We sampled stream sites during baseflow conditions between June and September of 2018 (*n* = 9) and 2019 (*n* = 11). We identified 75‐m sample reaches at each site and used two‐pass backpack electrofishing techniques (Smith‐Root LR24) with one electrofishing unit for each 3–4 m of stream width (Heimbuch et al., 1997). Fishes captured during each pass were placed in live wells, counted, identified to species, and returned to the stream. Fish unable to be identified in situ were euthanized with tricaine methanesulfonate and transported to the laboratory for identification. We estimated fish species abundance as the sum from the two electrofishing passes for each site. Fish were collected following U.S. National Park Service IACUC‐approved standard operating procedures.

### 2.2 Quantifying life history strategies

We compiled data on species life history traits to account for major sources of variation observed across North American freshwater fishes (Mims et al., 2010; Winemiller & Rose, 1992): maximum total body length (cm), spawning season length (months per year), age of female maturation (years), mean longevity (years), and fecundity (number of eggs per breeding female). We quantified parental care on an ordinal scale following Grabowska and Przybylski (2015): (1) nonguarding species that do not select spawning substrates, (2) nonguarding species that hide their broods, (3) guarding species that select spawning substrates, and (4) guarding species that spawn in nests. Species life history data were compiled from Jenkins and Burkhead (1994) and Frimpong and Angermeier (2009). We also classified species as native or introduced from Jenkins and Burkhead (1994). Species life history data are given in Table A2 in Appendix 2.

We used nonmetric multidimensional scaling (NMS) and archetype analysis (AA) to quantify life history strategies for each observed species. First, we fit a 2‐dimensional NMS ordination to log(*x*+1)‐transformed traits data with Bray–Curtis distances. Alternative distance measures (Euclidean, Gower) produced similar results as Bray–Curtis distances (results not shown). Second, we used AA to quantify species locations within the trilateral continuum defined by opportunistic, periodic, and equilibrium‐based endpoints (i.e., archetypes) following Pecuchet et al. (2017). AA is a technique used to quantify the location of observations in multidimensional space from their distance to extreme points (Cutler & Breiman, 1994), yielding a proportional life history strategy score for each species in our analysis. This approach is conceptually appropriate because most fish species exhibit some combination of life history strategies rather than a single strategy (Hitt et al., 2020; King & McFarlane, 2003; McManamay et al., 2015; Mims & Olden, 2012).

We then summed life history strategy scores from species presence/absence data for each site and scaled the cumulative site scores from 0 to 1 (Mims & Olden, 2012; Olden & Kennard, 2010; Pecuchet et al., 2017). This provided an index of the relative importance of opportunistic, periodic, and equilibrium‐based life history strategies at each sampling site for use in statistical models described below. We used R package “vegan” version 2.5‐7 (Oksanen et al., 2020) for NMS analysis and R package “archetypes” version 2.2‐0.1 (Eugster & Leisch, 2009) for AA.

### 2.3 Linking life history and environmental conditions

We compiled six environmental variables, including attributes of habitat volume, land use, karst terrain, and soil type (Table 1). We estimated site elevation and upstream basin size using LiDAR‐derived digital elevation models (1‐m resolution) with the USGS StreamStats Batch Processing Tool version 5.03 (USGS, 2021). We calculated the percent of urban land cover and agricultural land cover in upstream watersheds from the 2019 National Land Cover Dataset (NLCD) (Yang et al., 2018). Urban land cover was calculated as the sum of all “developed” NLCD classes (categories 21, 22, 23, 24), and agricultural land cover included hay/pasture, cultivated crops, and shrubland classes (categories 52, 71, 81, 82). We calculated the percent carbonate bedrock (i.e., limestone and dolomite) within each watershed using a national karst atlas (Weary & Doctor, 2014) to index potential effects of groundwater discharge on stream temperature and flow. We also used the STATSGO2 dataset (NRCS, 2021) to quantify the percent of soils in each watershed with the lowest infiltration rates and highest runoff potential (i.e., class D soils) (NRCS, 2007). Several highly correlated variables (Pearson *r* > .7) were excluded from further analysis (e.g., percent forest cover inversely related to percent agricultural land cover).

**TABLE 1:** Environmental covariates for sample sites: elevation (ELE), upstream basin area (UBA), percent urban land cover (URB), percent agricultural land cover (AGR), percent limestone parent material in karst terrain (KAR), and percent soil class D (SCD)

Site code	Site name	ELE (m)	UBA (ha)	URB (%)	AGR (%)	KAR (%)	SCD (%)
1	UNT to North Branch Potomac River	167	167	9.3	4.6	0.0	0.1
2*	UNT to Potomac River at Lock 71	173	2701	6.0	12.9	54.8	1.3
3	Town Creek	156	40,647	4.4	13.1	19.4	0.8
4*	UNT to Potomac River at Lock 62	151	130	1.6	0.5	0.0	0.0
5*	UNT to Potomac River at Lock 61	170	281	8.4	4.0	0.0	0.0
6	Sideling Hill Creek	144	26,916	6.1	18.2	0.0	0.3
7*	UNT to Potomac River	142	846	1.9	8.4	0.0	1.5
8*	UNT to Potomac River	140	503	6.6	24.4	55.2	0.7
9	Little Tonoloway Creek	127	6526	8.7	19.1	14.7	1.9
10	Tonoloway Creek	131	29,513	6.5	28.7	6.1	1.8
11	Green Spring Run	117	909	10.7	10.3	49.8	0.9
12	Little Conococheague Creek	112	4678	7.6	43.8	69.4	1.0
13*	UNT to Potomac River	92	284	13.7	70.4	100.0	0.0
14	Israel Creek	78	3370	8.5	33.0	0.0	2.1
15	Catoctin Creek	73	31,207	12.6	48.5	0.0	1.2
16*	Lander Branch	77	513	7.7	41.0	0.0	1.6
17	Tuscarora Creek	70	5463	14.3	65.7	25.3	1.8
18*	UNT to Potomac River	70	693	6.4	55.2	0.0	9.7
19*	UNT to Potomac River	66	182	1.3	39.1	0.0	5.6
20	Great Seneca Creek	57	33,557	34.7	31.0	0.0	4.6

Note

Percent data are given as the percent of upstream watershed areas. Site codes area mapped in Figure 1. Unnamed tributaries are abbreviated UNT. Sites codes with * were sampled during 2018; Otherwise sites were sampled in 2019. Site location coordinates are given in Table A1 in Appendix 1.

We evaluated environmental predictors of life history strategy scores among sites from beta regression models with AIC corrected for small sample size (AICc). Beta regression is appropriate for this analysis because the response variables (life history scores) are expressed as proportional data within sites (Douma & Weedon, 2019). We scaled environmental predictors to a mean of 0 and standard deviation of 1 to facilitate comparison of model coefficients. We then fit models using logit links for all additive combinations of environmental covariates (64 models per response variable). We evaluated AICc to rank the best models based on maximum likelihood estimation, and we considered models within 2.0 AICc units from the best model (ΔAICc) to be insignificantly different from one another (Burnham & Anderson, 2002). We used the R package “betareg” version 3‐1.4 to fit beta regression models (Cribari‐Neto & Zeileis, 2010) and R package “MuMIn” version 1.43.17 (Barton, 2020) to facilitate model comparisons.

We also used NMS to visualize environmental relationships with fish community composition among sites and physiographic regions. We fit a 2‐dimensional NMS ordination to log(*x*+1)‐transformed fish abundance with Bray–Curtis distances. We then plotted environmental covariates as vectors in the ordination space and evaluated their fit to the data after 1000 permutations. We used functions “metaMDS” and “envfit” in R package “vegan” version 2.5‐7 (Oksanen et al., 2020) for NMS analyses. We conducted all analyses in R version 4.1.1 (R Core Team, 2021).

## 3 RESULTS

**FIGURE A1:**
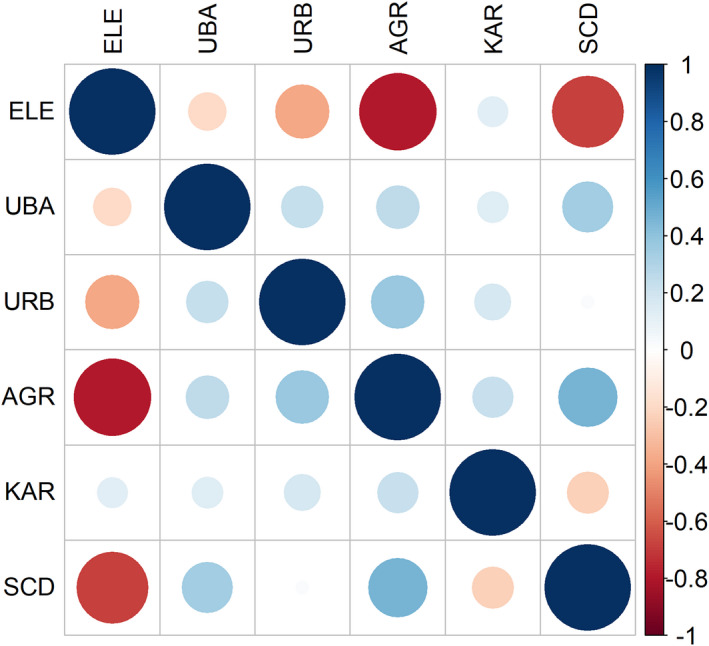
Spearman correlations in environmental variables across sites. Variables are abbreviated as elevation (ELE), upstream basin area (UBA), urban land cover (URB), agricultural land cover (AGR), carbonate parent material in karst terrain (KAR), and class D soils (SCD). Land cover and geological variables are expressed as the percent of upstream basin area.

Sample sites ranged in elevation 57–173 m above sea level (NAVD 88) with mean 116 m ± 9 m standard error (SE). Upstream basin areas ranged 130–40,647 ha with mean 9454 ha ± 3031 ha SE (Table 1). Agriculture was the primary nonforest land use (mean 29% of watershed area), followed by urban land cover (mean 9% of watershed area) (Table 1). Agricultural and urban land cover were positively correlated (Figure A1 in Appendix 3) but not monotonically related. For example, the eastern‐most site near Washington, D.C., showed the most extensive urbanization (Great Seneca Creek, site 20), whereas the greatest agricultural land cover was near the geographic center of the study area (an unnamed tributary near Shepherdstown WV, site 13). Class D soils (i.e., highest runoff potential) ranged from 0 to 10% of sampled watersheds, and the percent watershed area with carbonate bedrock (i.e., karst terrain) ranged from 0 to 100% (Table 1). Karst terrain included areas defined by the Oriskany formation (western Ridge and Valley region), the Keyser and Tonoloway formations (central Ridge and Valley region), and the Conococheague formation (eastern Ridge and Valley region) (Weary & Doctor, 2014). Carbonate bedrock was not strongly associated with other environmental covariates in the analysis (Spearman *r* < |.3|, *p* > .2, respectively) (Figure A1 in Appendix 3).

We collected 3801 individuals from 51 species of which 32 species (63%) were considered native to the Potomac River basin (Table 2). Species richness in sampled streams ranged from 1 to 25 (mean 17 ± 2 species SE), and abundance ranged from 11 to 764 individuals (mean 190 ± 34 individuals SE) (Table A3 in Appendix 4). Among taxonomic families, Leuciscidae contained the greatest richness with 19 species and nearly 50% of total abundance, followed by Centrarchidae with 11 species that constituted approximately 25% of all collected individuals. Percids and catostomids each were represented by 5 species, constituting 8% and 3% of total abundance, respectively. Ictalurids included 4 species, and Cottidae was represented by 3 species (Table 2). Remaining families were represented by a single species: American eel (Anguillidae: *Anguilla rostrata*), banded killifish (Fundulidae: *Fundulus diaphanus*), eastern mosquitofish (Poeciliidae: *Gambusia holbrooki*), and rainbow trout (Salmonidae: *Oncorhynchus mykiss*).

**TABLE 2:** Fish species abundance and occurrence observed during 2018–2019 in the study area (Figure 1)

Family	Species	Common name	Total abundance (% of total)	Mean abundance per site (SE)	Count of occupied sites (% of total)
Anguillidae	Anguilla rostrata* (ANRO)	American eel	19 (0.5)	1.0 (2.4)	6 (30)
Catostomidae	Catostomus commersoni* (CACO)	White sucker	83 (2.2)	4.2 (4.7)	16 (80)
	Erimyzon oblongus* (EROB)	Creek chubsucker	10 (0.3)	0.5 (1.1)	5 (25)
	Hypentelium nigricans* (HYNI)	Northern hogsucker	6 (0.2)	0.3 (1.1)	2 (10)
	*Moxostoma erythrurum* (MOER)	Golden redhorse	14 (0.4)	0.7 (2.4)	3 (15)
	Moxostoma macrolepidotum* (MOMA)	Shorthead redhorse	1 (<0.1)	0.1 (0.2)	1 (5)
Centrarchidae	*Ambloplites rupestris* (AMRU)	Rock bass	25 (0.7)	1.3 (2.8)	9 (45)
	Lepomis auritus* (LEAU)	Redbreast sunfish	68 (1.8)	3.4 (5.9)	9 (45)
	*Lepomis cyanellus* (LECY)	Green sunfish	443 (11.7)	22.2 (32.6)	18 (90)
	Lepomis gibbosus* (LEGI)	Pumpkinseed sunfish	20 (0.5)	1.0 (2.1)	6 (30)
	*Lepomis gulosus* (LEGU)	Warmouth	6 (0.2)	0.3 (1.3)	1 (5)
	*Lepomis macrochirus* (LEMA)	Bluegill	303 (8.0)	15.2 (32.1)	13 (65)
	*Lepomis megalotis* (LEME)	Longear sunfish	34 (0.9)	1.7 (4.6)	6 (30)
	*Lepomis microlophus* (LEMI)	Redear sunfish	4 (0.1)	0.2 (0.9)	1 (5)
	*Micropterus dolomieu* (MIDO)	Smallmouth bass	22 (0.6)	1.1 (1.5)	10 (50)
	*Micropterus salmoides* (MISA)	Largemouth bass	26 (0.7)	1.3 (2.7)	6 (30)
	*Pomoxis nigromaculatus* (PONI)	Black crappie	1 (<0.1)	0.1 (0.2)	1 (5)
Cottidae	Cottus caeruleomentum* (COCA)	Blue Ridge sculpin	15 (0.4)	0.8 (1.7)	5 (25)
	Cottus girardi* (COGI)	Potomac sculpin	154 (4.1)	7.7 (10.6)	10 (50)
	*Cottus*sp. cf. girardi* (COSP)	Checkered sculpin	96 (2.5)	4.8 (18.8)	2 (10)
Fundulidae Ictaluridae	Fundulus diaphanus* (FUDI)	Banded killifish	11 (0.3)	0.6 (1.4)	5 (25)
	Ameiurus natalis* (AMNA)	Yellow bullhead	233 (6.1)	11.7 (12.6)	15 (75)
	*Ictalurus punctatus*(ICPU)	Channel catfish	11 (0.3)	0.6 (1.3)	4 (20)
	Noturus insignis* (NOIN)	Margined madtom	35 (0.9)	1.8 (3.9)	6 (30)
	*Pylodictis olivaris* (PYOL)	Flathead catfish	8 (0.2)	0.4 (1.2)	3 (15)
Leuciscidae	Campostoma anomalum* (CAAN)	Central stoneroller	154 (4.1)	7.7 (10.8)	11 (55)
	*Carassius auratus* ^+^ (CAAU)	Goldfish	2 (0.1)	0.1 (0.3)	2 (10)
	Clinostomus funduloides* (CLFU)	Rosyside dace	23 (0.6)	1.2 (2.9)	5 (25)
	Cyprinella analostana* (CYAN)	Satinfin shiner	3 (0.1)	0.2 (0.7)	1 (5)
	Cyprinella spiloptera* (CYSP)	Spotfin shiner	132 (3.5)	6.6 (16.9)	8 (40)
	Exoglossum maxillingua* (EXMA)	Cutlip minnow	8 (0.2)	0.4 (1.1)	4 (20)
	Luxilus cornutus* (LUCO)	Common shiner	22 (0.6)	1.1 (2.6)	5 (25)
	*Nocomis leptocephalus* ^a^ (NOLE)	Bluehead chub	64 (1.7)	3.2 (8.1)	3 (15)
	Nocomis micropogon* (NOMI)	River chub	103 (2.7)	5.2 (15.1)	9 (45)
	Notemigonus crysoleucas* (NOCR)	Golden shiner	30 (0.8)	1.5 (3.4)	7 (35)
	Notropis buccatus* (NOBU)	Silverjaw minnow	12 (0.3)	0.6 (1.5)	4 (20)
	Notropis hudsonius* (NOHU)	Spottail shiner	142 (3.7)	7.1 (16.8)	7 (35)
	Notropis rubellus* (NORU)	Rosyface shiner	4 (0.1)	0.2 (0.7)	2 (10)
	*Notropis volucellus* (NOVO)	Mimic shiner	6 (0.2)	0.3 (1)	2 (10)
	Pimephales notatus* (PINO)^b^	Bluntnose minnow	401 (10.5)	20.1 (55.3)	16 (80)
	Rhinichthys atratulus* (RHAT)	Blacknose dace	398 (10.5)	19.9 (52.4)	11 (55)
	Rhinichthys cataractae* (RHCA)	Longnose dace	94 (2.5)	4.7 (7.4)	8 (40)
	Semotilus atromaculatus* (SEAT)	Creek chub	202 (5.3)	10.1 (15.3)	12 (60)
	Semotilus corporalis* (SECO)	Fallfish	38 (1.0)	1.9 (4.2)	7 (35)
Percidae	*Etheostoma blennioides* ^+^ (ETBL)	Greenside darter	109 (2.9)	5.5 (9.7)	9 (45)
	*Etheostoma caeruleum* (ETCA)	Rainbow darter	38 (1.0)	1.9 (3.3)	8 (40)
	Etheostoma flabellare* (ETFL)	Fantail darter	99 (2.6)	5.0 (5.9)	12 (60)
	Etheostoma olmstedi* (ETOL)	Tessellated darter	63 (1.7)	3.2 (5.5)	10 (50)
	*Sander vitreus* ^+^ (SAVI)	Walleye	1 (<0.1)	0.1 (0.2)	1 (5)
Poeciliidae	Gambusia holbrooki* (GAHO)	Eastern mosquitofish	4 (0.1)	0.2 (0.9)	1 (5)
Salmonidae	*Oncorhynchus mykiss* (ONMY)	Rainbow trout	1 (<0.1)	0.1 (0.2)	1 (5)

Note

Standard error (SE) for abundance across sites is given in parentheses. Native species are indicated with an asterisk (Jenkins & Burkhead, 1994) with 2 exceptions as indicated in superscripts. Species codes are plotted in Figure 3, and species traits data are given in Table A2 in Appendix 2.

^a^ Jenkins and Burkhead (1994) classify *N. leptocephalus* “native but possibly introduced,” and we consider it introduced.

^b^ Jenkins and Burkhead (1994) classify *P. notatus* “introduced but possibly native,” and we consider it native.

**FIGURE A2:**
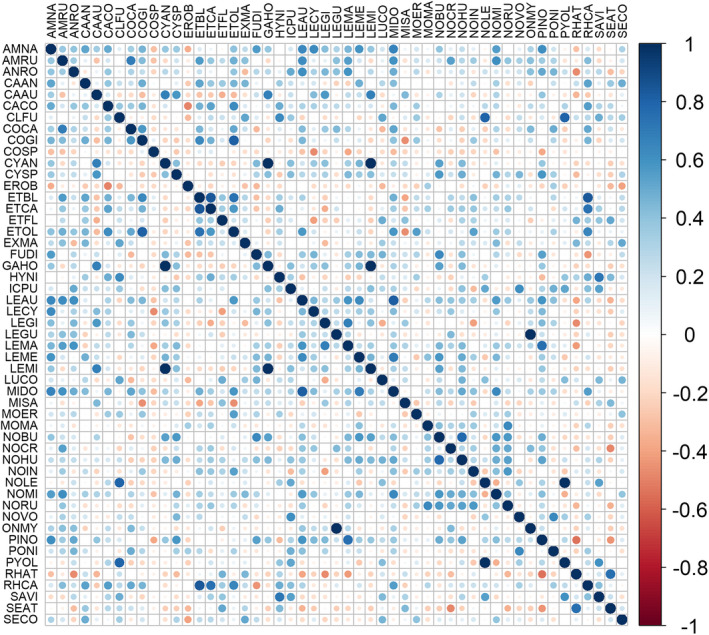
Spearman correlations in species abundance across sites. Species codes are given in Table 2 and Table A1 in Appendix 1

Green sunfish (*Lepomis cyanellus*), bluntnose minnow (*Pimephales notatus*), and blacknose dace (*Rhinichthys atratulus*) were the most abundant species in the dataset, comprising 12%, 11%, and 11% of total abundance, respectively (Table 2). Green sunfish was also the most widely distributed species, occurring in 90% of the sample sites. Bluntnose minnow and white sucker (*Catostomus commersoni*) were the second‐most widely distributed species, each occurring in 80% of the sites, followed by yellow bullhead (*Ameiurus natalis*) with occurrences in 75% of the sites (Table 2). Conversely, several species contained a single individual in the dataset: shorthead redhorse (*Moxostoma macrolepidotum*), black crappie (*Pomoxis nigromaculatus*), walleye (*Sander vitreus*), and rainbow trout. Cottid species included an undescribed sculpin (checkered sculpin, *Cottus* sp. cf. *girardi*) endemic to karst groundwater‐dominated streams in the Ridge and Valley portion of the Potomac River basin (Albertson, 1995; Hitt et al., 2021; Welsh, 1996). Checkered sculpin was the only species observed in one site (site 13; Figure 1) and was negatively associated with abundance of other species in the dataset (Figure A2 in Appendix 5).

**FIGURE 2:**
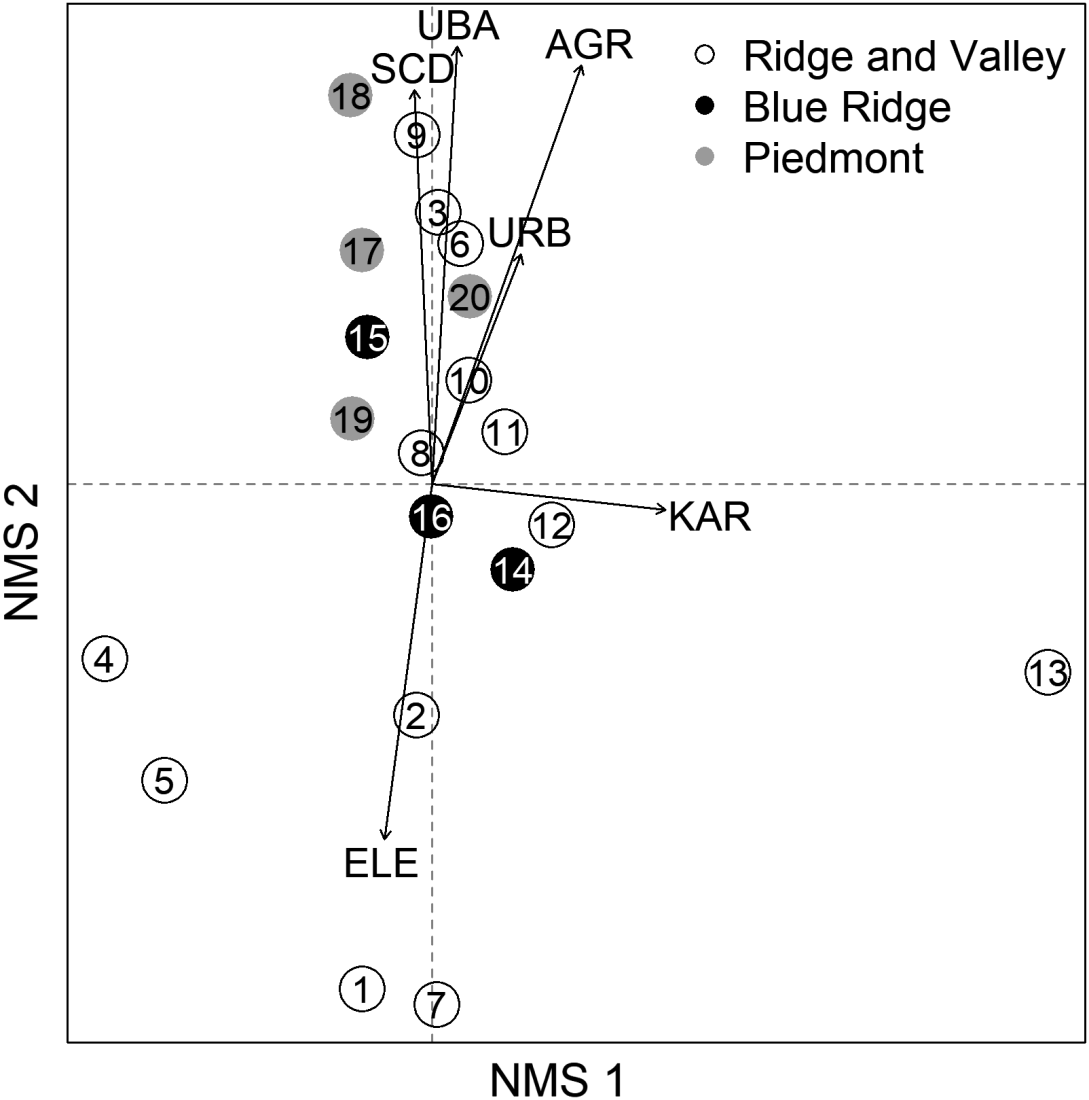
Nonmetric multidimensional scaling (NMS) ordination representing fish community structure by sites and physiographic regions. Site codes are given in Table 1, and environmental variables are represented by vectors for elevation (ELE), upstream basin area (UBA), urban land cover (URB), agricultural land cover (AGR), karst terrain (KAR), and soils with high runoff potential (SCD)

Spatial variation in fish community structure was represented by a 2‐dimensional NMS ordination of fish abundance data (stress = 0.10; Figure 2). Axis 1 was primarily associated with karst geology (axis loading = 1.0; Table 3) but also corresponded to agricultural and urban land cover (axis loadings = 0.63 and 0.64, respectively; Table 3). By contrast, variation along axis 2 primarily was defined by elevation, watershed area, and class D soils (axis loadings > |0.96|, respectively; Table 3). Physiographic regions varied primarily along axis 2, and fish communities in the Ridge and Valley region exhibited more spatial variation than communities from other physiographic regions (Figure 2).

**TABLE 3:** Covariate relationships to nonmetric multidimensional scaling (NMS) ordinations for fish assemblage structure (Figure 2) and life history strategy (Figure 3)

Model	Covariate	NMS axis 1	NMS axis 2	*R* ^2^	*p*
Fish assemblage structure	ELE	−0.287	−0.958	.246	.081
	UBA	0.129	0.992	.348	.018
	URB	0.656	0.755	.166	.228
	AGR	0.624	0.781	.514	.003
	KAR	0.999	−0.049	.489	.009
	SCD	−0.101	0.995	.281	.079
Life history strategy	TL	−0.996	−0.093	.503	<.005
	SS	0.630	0.777	.755	<.005
	MA	−0.953	−0.304	.636	<.005
	LO	−0.987	−0.162	.487	<.005
	FE	−0.921	0.389	.373	<.005
	PC	0.410	−0.912	.771	<.005

Note

Covariates are defined in Table 1 (fish assemblage structure) and Table 4 (life history strategy). Goodness of fit is indexed by the squared correlation coefficient (*R* ^2^) and empirical type‐1 error rate (*p*) from 1000 permutation tests.

Life history traits exhibited substantial variation among study species (Table 4; Table A2 in Appendix 2). Maximum adult body size ranged from 4 to 155 cm total length with the smallest species including eastern mosquitofish (4 cm), fantail darter (*Etheostoma flabellare*, 8 cm), and mimic shiner (*Notropis volucellus*, 8 cm) and the largest species including flathead catfish (*Pylodictus olivaris*, 155 cm), American eel (154 cm), and channel catfish (*Ictalurus punctatus*, 132 cm). Fecundity ranged from 134 eggs per female in Potomac sculpin (*Cottus girardi*) to over 10^6^ eggs per female in American eel, a catadromous species (Table 4; Table A2 in Appendix 2). Eastern mosquitofish matured at the youngest age (0.3 years) and exhibited the shortest lifespan (1 year) while American eel matured at 7 years and exhibited a mean lifespan of 25 years (Table 4; Table A2 in Appendix 2). The dataset included 16 species with the lowest level of parental care (nonguarding species that do not select spawning substrate) and 23 species with the highest level of parental care (guarding species that spawn in nests) (Table A2 in Appendix 2).

**TABLE 4:** Summary of life history variables across fish species (*n* = 51)

Summary	TL	SS	MA	LO	FE	PC
Minimum	4.0	0.5	0.3	1.0	134	1.0
Maximum	155.0	8.0	7.0	25.0	1,050,000	4.0
Mean	39.6	2.7	2.2	7.1	60,522	2.6
Standard deviation	37.2	1.6	1.1	4.7	172,736	1.3

Note

Variables are maximum total length (TL) in cm, annual spawning season length (SS) in months, female maturation age (MA) in years, longevity (LO) in years, fecundity (FE) in eggs/female, and parental care (PC) indexed on an ordinal scale from 1–4 (see text). Species life history data are given in Table A2 in Appendix 2.

**FIGURE 3:**
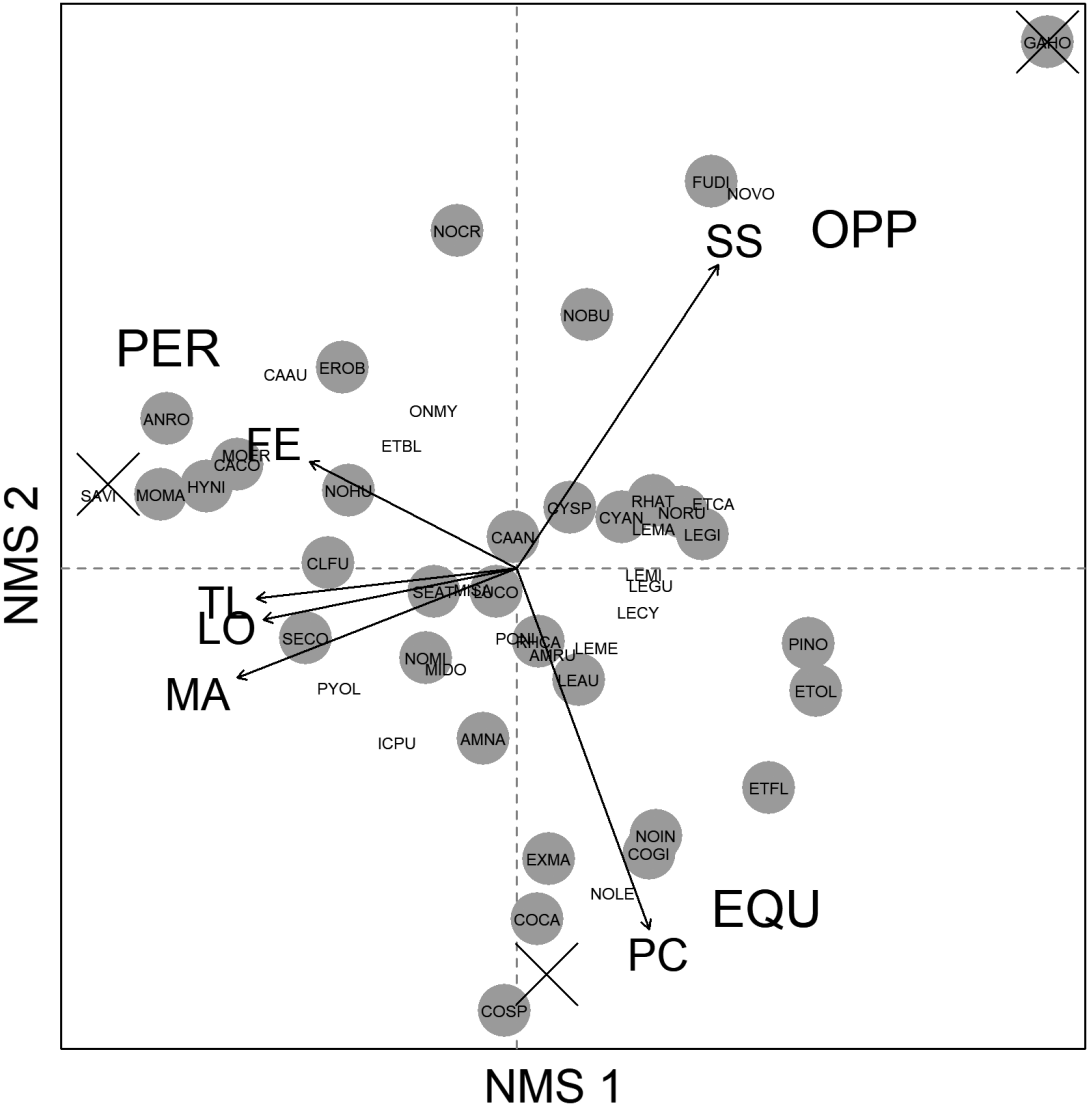
Nonmetric multidimensional scaling (NMS) ordination representing fish life history diversity. Variables are represented as vectors for spawning season length (SS), fecundity (FE), longevity (LO), total length (TL), female maturation age (MA), and parental care (PC). Archetype analysis endpoints associated with periodic (PER), equilibrium (EQU), and opportunistic (OPP) strategies are shown as “X.” Filled circles indicate native species. Species codes are given in Table 2, and life history data are given in Table A2 in Appendix 2

A 2‐dimensional NMS ordination represented interspecific variation in life history strategies (stress = 0.08; Figure 3). Axis 1 primarily indicated variation in body size and associated traits (fecundity, maturation age, longevity), and axis 2 primarily indicated a gradient between spawning season length and parental care (Table 3). Use of species NMS scores in AA identified 3 end‐member conditions (archetypes) representing opportunistic, periodic, and equilibrium life history strategies (Figure 3). Opportunistic strategies were typified by eastern mosquitofish (GAHO), which exhibit an extended spawning season, small body size, and low parental care. Periodic species were typified by walleye (SAVI), which exhibit large adult body size, high fecundity, late maturation, and long lifespan. Equilibrium species were characterized by checkered sculpin (COSP), which exhibit a short spawning season, small body size, and high parental care. Introduced species exhibited a range of opportunistic and periodic strategies, but only native species occupied the extreme equilibrium strategist space (Figure 3).

**FIGURE 4:**
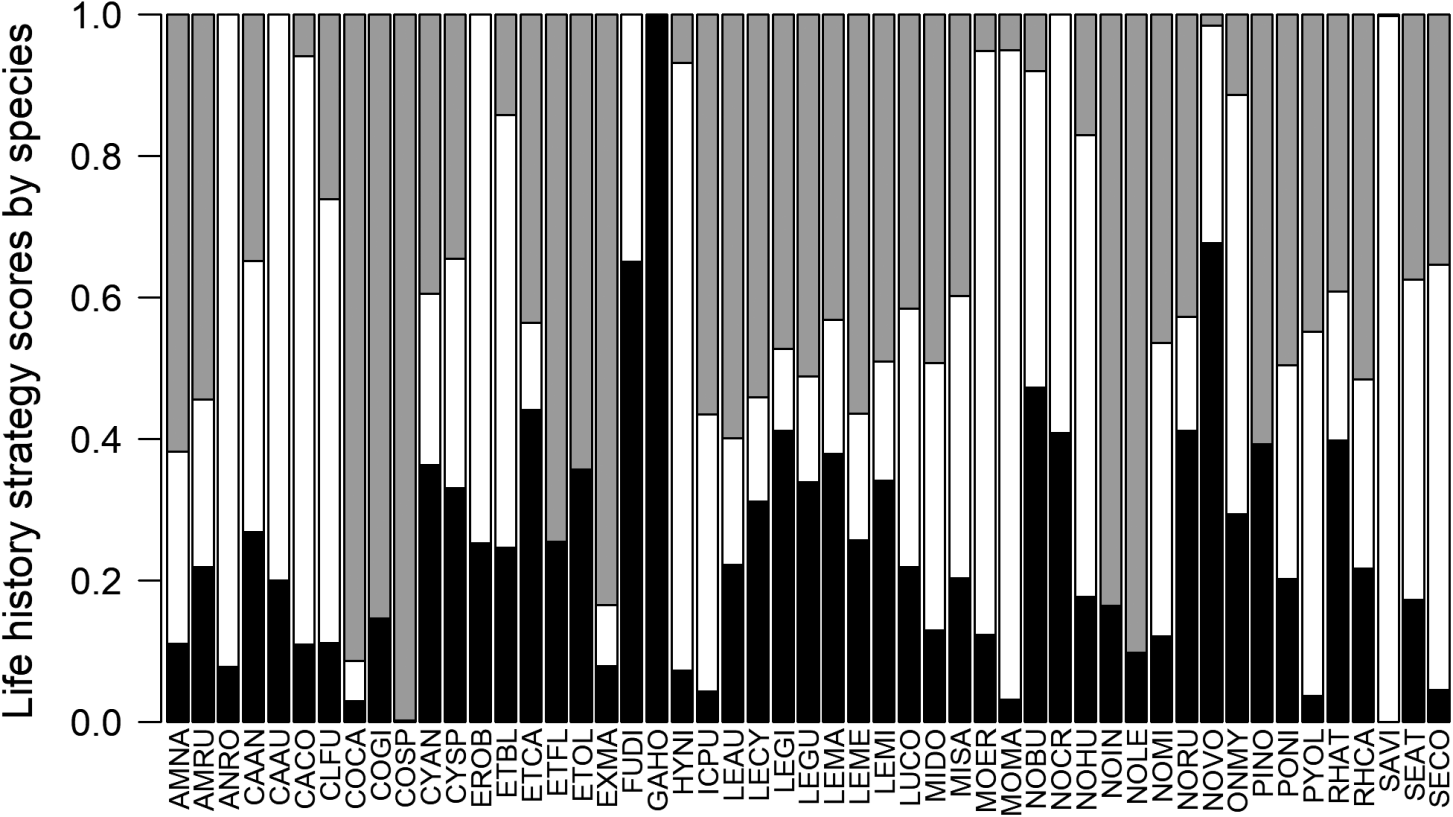
Species life history strategy scores from Archetype Analysis of life history traits (Figure 3) representing opportunistic (black), periodic (white), and equilibrium (grey) endpoints. Species codes are given in Table 2

Most species represented intermediate locations within the life history ordination space (Figure 3), and this was similarly reflected in species life history strategy scores derived from AA (Figure 4). End‐member species showed scores near 1.0 for opportunistic, periodic, and equilibrium‐based strategies, indicating a life history strategy nearly entirely defined as opportunistic (GAHO), periodic (SAVI), or equilibrium (COSP) (Figure 4). Other species were characterized by a mix of two or three strategies. For example, central stoneroller (*Campostoma anomalum*; CAAN) was located near the center of the life history space (Figure 3) and scored nearly evenly for each of the three strategies (Figure 4). By contrast, some species exhibited a combination of two of the three strategies: Golden shiner (*Notemigonus crysoleucas*; NOCR) was defined as a mix of opportunistic and periodic strategies but not equilibrium strategies (Figure 4) given its location in the NMS life history space (Figure 3). Alternatively, margined madtom (*Noturus insignis*; NOIN) showed a mix of opportunistic and equilibrium strategies but not periodic strategies (Figure 4).

Life history strategies were more variable within some taxonomic families than others. Each of the three cottid species in our analysis were characterized as strong equilibrium strategists (>85% equilibrium), and the five catostomid species were characterized by periodic strategies (>75% periodic; Figure 4). By contrast, other families exhibited more variation among species. Among percid darters (*Etheostoma* sp.), species varied primarily by trade‐offs between periodic and equilibrium scores. For example, greenside darter (*Etheostoma blennioides*; ETBL) showed a larger periodic strategy score (61%) than fantail darter (ETFL) or tessellated darter (*Etheostoma olmstedi*; ETOL) (<1%; Figure 4). Within Centrarchidae, species showed more variation in opportunistic and periodic strategies than equilibrium strategies. For example, although equilibrium scores were near 50% in both cases, smallmouth bass (*Micropterus dolomieu*; MIDO) represented a greater proportion of periodic strategy than opportunistic strategy, whereas pumpkinseed sunfish (*Lepomis gibbosus*; LEGI) showed the opposite pattern (Figure 4). Leuciscids exhibited a large range of life history strategies among species. For instance, mimic shiner (NOVO) and cutlip minnow (*Exoglossum maxillingua*; EXMA) showed inverse trends for opportunistic and equilibrium strategies (Figure 4): The former was characterized as an opportunistic strategist (68% opportunistic score), while the latter was characterized as an equilibrium strategist (84% equilibrium score).

**FIGURE 5:**
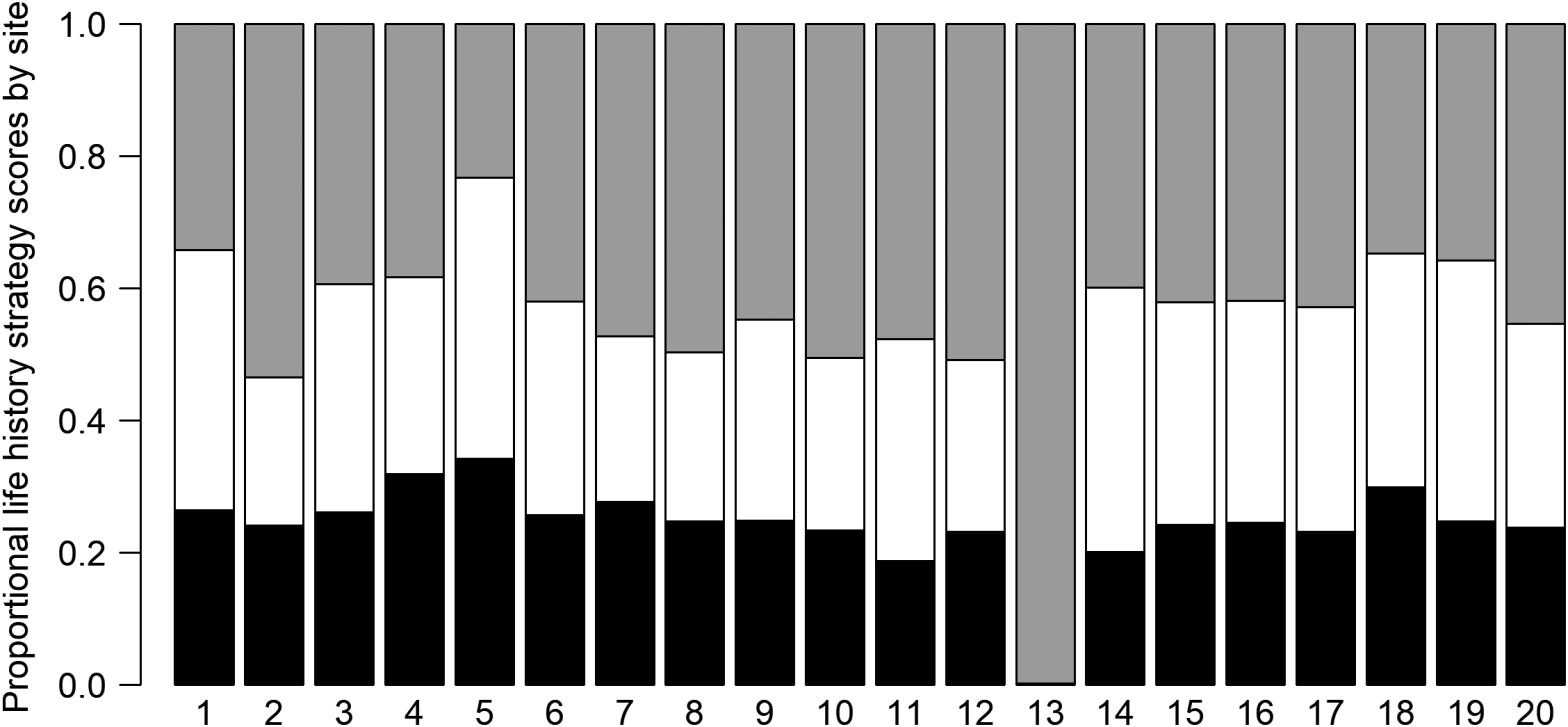
Proportional life history strategy scores across sites representing the abundance of opportunistic (black), periodic (white), and equilibrium (gray) strategists. Site codes are given in Table 1

Site‐level life history scores calculated as the proportional sum of strategy scores for observed species exhibited a mix of opportunistic, periodic, and equilibrium strategies for nearly all sites (Figure 5). The sole exception was site 13 where only a strong equilibrium strategist species was observed (checkered sculpin). Of the other 19 sites, the proportional score for opportunistic strategies ranged from 0.19 to 0.34 with a mean of 0.24, periodic strategies ranged from 0.22 to 0.42 with a mean of 0.31, and equilibrium strategies ranged from 0.23 to 0.53 with a mean of 0.45 (Figure 5). Equilibrium strategies comprised the largest share of life history scores among sites on average; however, periodic scores exceeded equilibrium scores in five of the 20 sites (Figure 5).

Beta regression models identified environmental predictors of fish life history strategies across sites (Table 5). The most parsimonious models for opportunistic strategies showed negative relationships with karst terrain and agriculture and positive relationships to elevation and class D soils, accounting for 44%–62% of the observed variation across sites. Periodic strategies also decreased with karst terrain, and no other terms were included in the best model which accounted for 51% of the observed variation (Table 5). By contrast, equilibrium strategies increased with karst terrain and agriculture and decreased with elevation in the best models which accounted for 52%–65% of the observation variation across sites. Among all models, karst terrain exhibited stronger effects than other covariates, as indicated by the greater absolute magnitude of standardized model coefficients (Table 5). Basin size and urbanization were not included in the best models for any life history strategy.

**TABLE 5:** Top models for environmental predictors of fish life history strategies across sites (*n* = 20)

Response variable	Model rank	Standardized beta regression coefficients						Model summary			
		ELE	UBA	URB	AGR	KAR	SCD	AICc	ΔAICc	AIC weight	*R* ^2^
% Opportunistic	1	0.46				−0.57	0.35	−41.65	0.00	0.30	.62
	2				−0.47	−0.36	0.32	−41.19	0.46	0.23	.61
	3				−0.24	−0.56		−40.06	1.59	0.13	.50
	4					−0.67		−39.92	1.73	0.12	.44
	5	0.21				−0.68		−39.83	1.82	0.12	.49
	6				−0.70		0.52	−39.37	2.28	0.09	.53
% Periodic	1					−0.55		−34.57	0.00	0.45	.51
	2				−0.10	−0.51		−32.17	2.40	0.13	.54
	3	0.07				−0.55		−31.82	2.76	0.11	.53
	4					−0.52	0.06	−31.74	2.83	0.11	.52
	5		0.05			−0.54		−31.70	2.87	0.11	.52
	6			−0.02		−0.54		−31.46	3.11	0.09	.51
% Equilibrium	1					0.71		−19.83	0.00	0.25	.52
	2				0.23	0.62		−19.45	0.38	0.21	.58
	3				0.39	0.48	−0.27	−19.02	0.81	0.17	.65
	4	−0.20				0.73		−18.92	0.91	0.16	.57
	5	−0.38				0.65	−0.31	−18.78	1.05	0.15	.65
	6			0.11		0.69		−17.27	2.56	0.07	.54

Note

Environmental variables are defined in Table 1. Excluded variables are indicated with a dash. AICc gives the Akaike information criterion corrected for small sample size. Models with ∆AICc < 2.0 were considered to share statistical support for the best model.

## 4 DISCUSSION

Our results indicate the utility of life history theory for understanding the ecological importance of environmental stability and stochasticity. First, we identified trade‐offs between fecundity, spawning season duration, and parental care that organized species along a trilateral continuum of opportunistic‐, periodic‐, and equilibrium‐type life history strategies, consistent with prior research (Hitt et al., 2020; McManamay et al., 2015; Mims & Olden, 2012; Winemiller & Rose, 1992). Second, we identified mechanistic effects of watershed hydrology: We showed that opportunistic life history strategies were more common where flashy runoff is expected and less common in karst terrain where groundwater inputs are expected to stabilize stream temperature and flow (Table 5). Prior research has demonstrated effects of flow regulation on life history diversity within riverine fish communities (Kominoski et al., 2018; McManamay & Frimpong, 2015; Mims & Olden, 2013; Olden et al., 2006; Perkin et al., 2017), and our study extends this perspective from regulated rivers into headwater streams. Our findings also suggest the utility of life history theory for understanding ecological responses to destabilized environmental conditions under global climate change.

The diversity of life history strategies we observed in the Potomac River basin was consistent with prior research at the continental scale (McManamay et al., 2015; Mims et al., 2010; Winemiller & Rose, 1992), indicating evolutionary processes that transcend zoogeographic boundaries. This is particularly noteworthy for the study area due to zoogeographic effects of Great Falls of the Potomac on fish species richness and endemism (Jenkins & Burkhead, 1994; Stauffer et al., 1995). For example, checkered sculpin, Potomac sculpin, and Blue Ridge sculpin (*Cottus caeruleomentum*) represented end‐members for the equilibrium strategy due to their low fecundity, small body size, and investment in parental care (Figure 3). This pattern has been shown previously for freshwater sculpins (Winemiller, 2005) even though the species in our analysis are endemic to the region (Hitt et al., 2021; Kinziger et al., 2000; Robins, 1961). Other species in our analysis maintain larger geographic distributions and showed concordant life history patterns with prior research. For example, Winemiller and Rose (1992) identified *Gambusia* sp. an exemplar of the opportunistic strategy, corresponding with our results.

Karst terrain was an important predictor of life history strategies (Table 5), indicating the importance of groundwater–surface water interactions for stream fish community composition. Groundwater depth and volume influence the thermal resiliency of stream ecosystems (Briggs et al., 2018; Hare et al., 2021; Johnson et al., 2020; Snyder et al., 2015), and streams located in karst terrain are strongly influenced by losses from the surface to aquifers and the emergence of groundwater through springs and seeps (Bonacci et al., 2009). Our analysis demonstrated that karst terrain was associated with a life history strategy that capitalizes on stable environmental conditions, suggesting a stabilizing effect of karst groundwater dynamics on stream fish habitat conditions.

**FIGURE A3:**
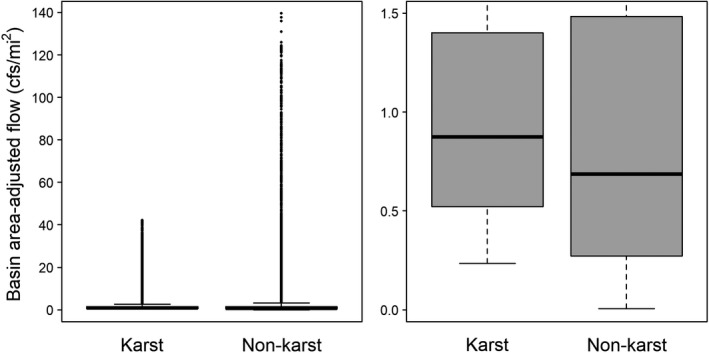
Comparison of stream flow variability in karst terrain vs nonkarst terrain within the study area. The karst site is located at Antietam Creek near Waynesboro, Pennsylvania (U.S. Geological Survey gage #01619000), and the nonkarst site is located at Catoctin Creek near Middletown, Maryland (U.S. Geological Survey gage #01637500). Flow data were adjusted for upstream basin area in each site (karst site = 93.5 mi^2^; nonkarst site = 66.9 mi^2^). The nonkarst site exhibited greater variance in basin area‐adjusted flow (Conover squared ranks test *p* < .0001) in this sample of over 497,000 observations from 7/1/2005 to 7/1/2020. This test statistic provides a nonparametric version of Levene's test for homogeneity of variance among groups

However, groundwater in karst terrain typically exhibits spatially and temporally complex flow and recharge dynamics rather than spatially uniform processes (Bonacci et al., 2009; Evaldi et al., 2009; Kozar et al., 1991), and this can affect streams in divergent ways. For instance, in their study of karst landscapes of the Ozark‐Ouachita highlands region, Leasure et al. (2016) classified stream flow types as “groundwater stable” or “groundwater flashy,” and Magoulick et al. (2021) attributed seasonal structure in stream fish community composition to these hydrological differences. Vesper and Herman (2020) also recognized differences between limestone and dolomite springs in the study area based on their chemical composition. We cannot fully account for potential differences among karst types in our study because most of the sampled streams lacked flow gages, and mainstem river gages typically underrepresent variation observed in headwater streams (Deweber et al., 2014; Kovach et al., 2019). However, one karst stream in our study area supports flow data (Antietam Creek, USGS gage 01619000), and flow in this site was less variable than in a nearby stream outside of karst terrain (Catoctin Creek, USGS gage 01637500) (Figure A3 in Appendix 6). This finding is consistent with prior research indicating the overriding importance of fractured rock layers for groundwater flow rather than conduits or caves within the study area (Evaldi et al., 2009; Kozar et al., 1991; White, 1977) because increased rock contact area facilitates conductive heat exchange processes and moderates quickflow storm responses (Bonacci et al., 2009).

Our study also indicates the importance of soil properties and runoff processes for fish life history strategies. In contrast to karst terrain, we found that streams draining watersheds with high runoff potential were associated with opportunistic life history strategists (Table 5), suggesting the importance of an extended spawning period and short generation time to facilitate recovery from repeated disturbance events (i.e., discrete high or low flow events; Resh et al., 1988). Schlosser (1990) observed longitudinal variation in opportunistic life history strategies and attributed this to flashy flows in headwater areas versus the comparative stability of larger rivers. In contrast, we found that basin size (i.e., an index of stream volume) was less important than soil type in our best models, suggesting an overriding effect of soil properties and runoff dynamics. Hydrologic soil classification data are available globally (Ross et al., 2018), and this provides opportunities to evaluate the patterns observed here within other zoogeographic and physiographic regions.

In contrast to our expectation, nonforest land use did not increase opportunistic life history strategies. Instead, agricultural development showed no relationship to opportunistic strategies and was positively associated with equilibrium strategies. Moreover, we found no effect of urbanization in the best models, despite evidence that impervious land cover increases downstream peak flows (Anderson, 1970; O'Driscoll et al., 2010; Sauer et al., 1983) and evidence that increasing peak flows promotes opportunistic life history strategies in Potomac River fish communities (Hitt et al., 2020). This result may be due to the spatial arrangement of our sample sites (see below) or due to moderating effects of karstic groundwater on stream ecosystem responses to land use practices. For example, Kollaus et al. (2015) attributed temporal stability of fish communities in an urbanizing landscape to moderating effects of karst terrain and associated groundwater processes.

Alternatively, our index of parental care may indicate avoidance of substrate embeddedness or other physical habitat alternations associated with agricultural development (Diana et al., 2006). For instance, bluehead chub (*Nocomis leptocephalus*; NOLE) exhibits high levels of parental care due to nest construction and maintenance, and this behavior enables population persistence in agricultural landscapes by clearing fine substrates from spawning areas (Hitt & Roberts, 2012; Peoples et al., 2011). We also observed this species in streams draining watersheds with extensive agricultural development (sites 14, 15, and 17), suggesting that parental care may compensate for potentially adverse environmental conditions. Likewise, checkered sculpin exhibits nest cleaning behaviors to remove fine sediments (R. Hagerty, U.S. Fish and Wildlife Service; personal communication), and this species was observed in sites with extensive agricultural development (sites 12 and 13; Table A3 in Appendix 4).

The spatial arrangement of sample sites has implications for the interpretation of our results. The sites encompassed a large range of environmental conditions (i.e., large and small streams within 3 physiographic regions), which facilitated generalizations about the observed environmental effects. However, site elevation was inversely correlated to the percent of soils with high runoff potential, and therefore we could not fully partition effects of air temperature or other attributes associated with site elevation versus runoff processes associated with soil type. In addition, site elevation was inversely related to the size of the Potomac River near stream confluences, so we could not partition effects of elevation from fish dispersal from riverine source populations (i.e., mass effects; Leibold et al., 2004). Dispersal from riverine source populations has been demonstrated in many zoogeographic regions (Gorman, 1986; Hitt & Angermeier, 2008, 2011; Osborne & Wiley, 1992; Paukert et al., 2006), and 8 of the 51 species in our dataset (16%) were previously classified as “riverine specialists” (Hitt & Angermeier, 2011): flathead catfish, walleye, channel catfish, mimic shiner, rosyface shiner (*Notropis rubellus*), greenside darter, longear sunfish (*Lepomis megalotis*), and spotfin shiner (*Cyprinella spiloptera*). Moreover, stream confluences can create unique physical habitat features (Benda et al., 2004), and we were unable to account for such potential effects in our sampling design.

Our use of AA demonstrated its utility for quantifying species life history as composites of disparate strategies, and this was appropriate given the mix of life history traits that most fishes exhibit (Hitt et al., 2020; King & McFarlane, 2003; Winemiller & Rose, 1992). However, our use of AA was facilitated by the large range of life history traits among species in our dataset which permitted us to interpret meaningful end‐members for each life history strategy. For instance, eastern mosquitofish represented the opportunistic strategy, and the absence of this species in the dataset would have established an opportunistic endpoint relative to banded killifish and mimic shiner rather than the more extreme traits of eastern mosquitofish. Applications of AA therefore can enable quantitative interpretation of species life history as combinations of strategies (Pecuchet et al., 2017) but require interpretation relative to patterns observed across large geographic regions (Mims et al., 2010; Winemiller & Rose, 1992).

A central tenet in climate change research is that biological responses will be more sensitive to extreme environmental conditions than average conditions (Turner et al., 2020), and our study indicates the utility of life history theory for understanding these mechanisms (Lancaster et al., 2017). Many river systems have shown increased flow variation over recent decades (Coumou & Rahmstorf, 2012; Milly et al., 2008; Rahmstorf & Coumou, 2011; Ward et al., 2015) in response to extreme precipitation events (Easterling et al., 2000; Gershunov et al., 2019). Prior research has demonstrated the importance of scouring flows for fish population dynamics (Blum et al., 2018; Kanno et al., 2015), and our study extends this perspective to the community level through the analysis of life history traits that transcend zoogeographic boundaries. Our results also suggest that groundwater processes in karst terrain stabilize environmental conditions in receiving streams, but the sensitivity of these systems will depend in part on groundwater depth (Hare et al., 2021), spatially complex flow pathways (Kozar et al., 1991), and temporal lags in precipitation response (Schreiner‐McGraw & Ajami, 2021) that control stream habitat resiliency to atmospheric change.

## CONFLICT OF INTEREST

The authors declare no conflicts of interest.

## AUTHOR CONTRIBUTIONS

**Nathaniel P. Hitt:** Conceptualization (equal); Formal analysis (lead); Investigation (lead); Methodology (lead); Writing – original draft (lead); Writing – review & editing (lead). **Andrew P. Landsman:** Conceptualization (equal); Data curation (lead); Formal analysis (supporting); Investigation (supporting); Writing – original draft (supporting); Writing – review & editing (supporting). **Richard L. Raesly:** Data curation (supporting); Methodology (supporting); Writing – original draft (supporting); Writing – review & editing (supporting).

### OPEN RESEARCH BADGES

This article has earned an Open Data Badge for making publicly available the digitally‐shareable data necessary to reproduce the reported results. The data is available at [https://irma.nps.gov/DataStore/Reference/Profile/2288619].

## Data Availability

Data are available from the U.S. National Park Service's Integrated Resource Management Applications data portal: (https://irma.nps.gov/DataStore/Reference/Profile/2288619).

## APPENDIX 1

**TABLE A1:** Sample site coordinates. Site locations are mapped in Figure 1. Unnamed tributaries are abbreviated UNT

Site code	Site name	Site coordinates in decimal degrees (NAD83)
1	UNT to North Branch Potomac River	39.538883, −78.650270
2	UNT to Potomac River at Lock 71	39.541512, −78.617686
3	Town Creek	39.523621, −78.544266
4	UNT to Potomac River at Lock 62	39.571003, −78.453110
5	UNT to Potomac River at Lock 61	39.584040, −78.459903
6	Sideling Hill Creek	39.639885, −78.334251
7	UNT to Potomac River	39.631583, −78.308974
8	UNT to Potomac River	39.651125, −78.242464
9	Little Tonoloway Creek	39.697782, −78.183025
10	Tonoloway Creek	39.697782, −78.157477
11	Green Spring Run	39.607730, −77.970462
12	Little Conococheague Creek	39.604222, −77.909207
13	UNT to Potomac River	39.430468, −77.764586
14	Israel Creek	39.328337, −77.683076
15	Catoctin Creek	39.311602, −77.569350
16	Lander Branch	39.303171, −77.557221
17	Tuscarora Creek	39.244011, −77.474710
18	UNT to Potomac River	39.193828, −77.470232
19	UNT to Potomac River	39.159348, −77.516609
20	Great Seneca Creek	39.090286, −77.329515

## APPENDIX 2

**TABLE A2:** Species traits data. Life history variables are maximum total length (TL) in cm, annual spawning season length (SS) in months, female maturation age (MA) in years, longevity (LO) in years, fecundity (FE) in eggs per female, and parental care (PC) indexed from 1 to 4 (see text). Data sources are Jenkins and Burkhead (1994) and Frimpong and Angermeier (2009)

Family	Species name	Code	Common name	TL	SS	MA	LO	FE	PC
Anguillidae	*Anguilla rostrata*	ANRO	American eel	152	2.7	7.0	25.0	1,050,000	1
Catostomidae	*Catostomus commersoni*	CACO	White sucker	64	1.8	3.0	8.0	50,000	1
	*Erimyzon oblongus*	EROB	Creek chubsucker	36	2.3	2.0	5.5	83,013	1
	*Hypentelium nigricans*	HYNI	Northern hogsucker	61	1.5	3.0	11.0	30,000	1
	*Moxostoma erythrurum*	MOER	Golden redhorse	78	2.0	3.5	8.0	23,350	1
	*Moxostoma macrolepidotum*	MOMA	Shorthead redhorse	75	1.3	3.5	12.0	44,000	1
Centrarchidae	*Ambloplites rupestris*	AMRU	Rock bass	43	3.0	3.0	8.0	11,000	4
	*Lepomis auritus*	LEAU	Redbreast sunfish	31	2.3	2.0	6.0	10,000	4
	*Lepomis cyanellus*	LECY	Green sunfish	31	4.0	2.0	8.0	10,000	4
	*Lepomis gibbosus*	LEGI	Pumpkinseed sunfish	40	7.0	2.0	8.0	14,000	4
	*Lepomis gulosus*	LEGU	Warmouth	31	3.8	1.5	8.0	63,000	4
	*Lepomis macrochirus*	LEMA	Bluegill	41	6.0	2.0	10.0	50,000	4
	*Lepomis megalotis*	LEME	Longear sunfish	24	2.5	2.0	7.0	22,119	4
	*Lepomis microlophus*	LEMI	Redear sunfish	43	4.0	2.0	5.0	80,000	4
	*Micropterus dolomieu*	MIDO	Smallmouth bass	69	2.3	3.5	15.0	27,000	4
	*Micropterus salmoides*	MISA	Largemouth bass	97	3.0	2.5	16.0	109,314	4
	*Pomoxis nigromaculatus*	PONI	Black crappie	49	2.3	2.5	8.0	188,000	4
Cottidae	*Cottus caeruleomentum* ^a^	COCA	Blue Ridge sculpin	15	1.3	2.0	6.0	176	4
	*Cottus girardi*	COGI	Potomac sculpin	14	2.0	2.0	5.0	134	4
	*Cottus*sp. cf. *girardi* ^b^	COSP	Checkered sculpin	9	0.5	2.0	5.0	689	4
Fundulidae	*Fundulus diaphanus*	FUDI	Banded killifish	10	6.0	1.0	4.0	252	1
Ictaluridae	*Ameiurus natalis*	AMNA	Yellow bullhead	47	1.5	2.5	7.0	7000	4
	*Ictalurus punctatus*	ICPU	Channel catfish	132	1.5	3.5	10.0	10,600	4
	*Noturus insignis*	NOIN	Margined madtom	15	2.0	2.0	4.0	223	4
	*Pylodictis olivaris*	PYOL	Flathead catfish	155	1.5	4.5	23.0	100,000	4
Leuciscidae	*Campostoma anomalum*	CAAN	Central stoneroller	22	2.5	2.5	5.0	4800	2
	*Carassius auratus*	CAAU	Goldfish	59	3.0	3.5	10.0	400,000	1
	*Clinostomus funduloides*	CLFU	Rosyside dace	11	1.0	2.0	4.0	560	1
	*Cyprinella.analostana*	CYAN	Satinfin shiner	11	2.8	1.5	4.0	3628	2
	*Cyprinella spiloptera*	CYSP	Spotfin shiner	12	2.8	2.0	5.0	7474	2
	*Exoglossum maxillingua*	EXMA	Cutlip minnow	16	1.0	2.0	4.5	1177	4
	*Luxilus cornutus*	LUCO	Common shiner	18	2.0	2.0	6.0	1950	2
	*Nocomis leptocephalus*	NOLE	Bluehead chub	26	1.5	1.5	2.5	800	4
	*Nocomis micropogon*	NOMI	River chub	32	1.5	2.0	5.0	1000	2
	*Notemigonus crysoleucas*	NOCR	Golden shiner	30	3.5	1.0	8.0	4700	1
	*Notropis buccatus*	NOBU	Silverjaw minnow	10	3.5	1.5	3.0	1762	1
	*Notropis hudsonius*	NOHU	Spottail shiner	15	1.3	1.5	4.5	3709	1
	*Notropis rubellus*	NORU	Rosyface shiner	9	3.0	1.5	3.0	1500	2
	*Notropis volucellus*	NOVO	Mimic shiner	8	6.5	1.0	3.0	1000	1
	*Pimephales notatus*	PINO	Bluntnose minnow	11	3.3	1.0	3.5	4195	4
	*Rhinichthys atratulus*	RHAT	Blacknose dace	10	3.0	1.5	3.5	2674	2
	*Rhinichthys cataractae*	RHCA	Longnose dace	22	2.0	2.5	5.0	10,000	3
	*Semotilus atromaculatus*	SEAT	Creek chub	30	1.5	2.0	5.0	7157	2
	*Semotilus corporalis*	SECO	Fallfish	51	1.0	2.5	9.0	12,000	2
Percidae	*Etheostoma blennioides*	ETBL	Greenside darter	17	1.8	1.5	5.0	2000	1
	*Etheostoma caeruleum*	ETCA	Rainbow darter	8	3.0	1.0	4.0	1462	2
	*Etheostoma flabellare*	ETFL	Fantail darter	8	2.0	1.0	4.0	467	4
	*Etheostoma olmstedi*	ETOL	Tessellated darter	11	3.0	1.0	3.0	1435	4
	*Sander vitreus*	SAVI	Walleye	90	1.0	3.0	14.0	600,000	1
Poeciliidae	*Gambusia holbrooki*	GAHO	Eastern mosquitofish	4	8.0	0.3	1.0	315	1
Salmonidae	*Oncorhynchus mykiss*	ONMY	Rainbow trout	120	4.0	4.0	7.0	27,000	2

^a^ Data from mottled sculpin (*Cottus bairdii*).

^b^ Data from slimy sculpin (*Cottus cognatus*).

## APPENDIX 4

**TABLE A3:** Species abundance by site. Site codes are given in Table 1, and species codes are given in Table 2

Species code	Site code																			
	1	2	3	4	5	6	7	8	9	10	11	12	13	14	15	16	17	18	19	20
AMNA	0	2	6	0	0	9	0	27	24	7	21	5	0	1	6	22	20	49	13	21
AMRU	0	1	3	0	0	1	0	0	13	2	1	0	0	0	1	1	0	0	0	2
ANRO	0	0	2	0	0	0	0	0	3	0	0	0	0	1	0	0	1	1	0	11
CAAN	0	0	0	0	0	2	0	31	0	21	31	26	0	7	2	8	0	2	7	17
CAAU	0	0	0	0	0	0	0	0	0	0	0	0	0	0	0	0	0	1	1	0
CACO	3	1	1	0	0	3	0	1	9	7	16	4	0	12	1	1	13	6	3	2
CLFU	0	0	0	0	0	0	0	0	0	0	2	0	0	12	6	1	2	0	0	0
COCA	0	1	0	0	0	0	0	0	3	2	2	0	0	0	0	0	0	0	0	7
COGI	0	2	9	0	0	5	0	19	13	11	28	38	0	0	0	0	10	0	0	19
COSP	0	0	0	0	0	0	0	0	0	0	0	10	86	0	0	0	0	0	0	0
CYAN	0	0	0	0	0	0	0	0	0	0	0	0	0	0	0	0	0	3	0	0
CYSP	1	0	7	0	0	2	0	0	0	0	0	0	0	0	8	4	0	76	23	11
EROB	0	0	0	4	3	1	1	0	0	0	0	0	0	0	0	0	0	0	0	1
ETBL	0	0	8	0	0	5	0	0	1	21	20	37	0	3	2	0	0	0	0	12
ETCA	0	0	2	0	0	6	0	0	2	2	6	13	0	6	1	0	0	0	0	0
ETFL	0	8	7	0	0	4	12	4	0	6	3	8	0	23	3	15	0	0	0	6
ETOL	0	0	3	0	0	3	0	1	1	14	20	1	0	1	0	0	8	0	0	11
EXMA	0	0	0	0	0	0	0	0	0	1	1	0	0	0	1	5	0	0	0	0
FUDI	0	0	1	0	0	0	0	1	0	0	0	0	0	0	0	6	1	2	0	0
GAHO	0	0	0	0	0	0	0	0	0	0	0	0	0	0	0	0	0	4	0	0
HYNI	0	0	0	0	0	0	0	0	0	0	1	0	0	5	0	0	0	0	0	0
ICPU	0	0	0	0	0	0	0	0	0	0	0	0	0	5	1	0	0	2	0	3
LEAU	0	0	6	0	0	7	0	1	14	10	0	0	0	0	0	1	2	4	0	23
LECY	7	3	19	4	70	5	0	40	39	21	14	2	0	5	7	12	21	144	22	8
LEGI	0	9	0	0	0	0	0	0	1	0	0	0	0	0	0	0	1	4	3	2
LEGU	0	0	0	0	0	0	0	0	6	0	0	0	0	0	0	0	0	0	0	0
LEMA	0	114	20	0	0	8	1	0	20	2	0	0	0	1	5	4	12	105	2	9
LEME	0	0	0	0	0	4	0	3	0	3	0	0	0	0	0	2	0	21	0	1
LEMI	0	0	0	0	0	0	0	0	0	0	0	0	0	0	0	0	0	4	0	0
LUCO	0	0	1	0	0	0	0	3	0	0	0	0	0	5	2	11	0	0	0	0
MIDO	0	0	1	0	0	2	0	1	3	6	1	0	0	1	0	1	0	3	0	3
MISA	0	7	0	0	0	0	1	0	0	0	0	0	0	2	0	10	0	1	5	0
MOER	0	0	2	0	0	0	0	0	0	0	11	0	0	0	0	0	1	0	0	0
MOMA	0	0	0	0	0	1	0	0	0	0	0	0	0	0	0	0	0	0	0	0
NOBU	0	0	1	0	0	2	0	0	0	0	0	0	0	0	0	3	0	6	0	0
NOCR	0	0	4	0	1	3	0	0	15	3	0	0	0	0	1	0	0	0	3	0
NOHU	0	0	38	0	0	28	0	0	0	1	0	0	0	5	1	3	0	66	0	0
NOIN	0	0	9	0	0	8	1	1	0	15	1	0	0	0	0	0	0	0	0	0
NOLE	0	0	0	0	0	0	0	0	0	0	0	0	0	25	28	0	11	0	0	0
NOMI	0	0	8	0	0	3	0	0	2	8	3	0	0	0	0	70	0	2	6	1
NORU	0	0	3	0	0	1	0	0	0	0	0	0	0	0	0	0	0	0	0	0
NOVO	0	0	0	0	0	0	0	0	0	0	0	0	0	0	4	0	0	0	0	2
ONMY	0	0	0	0	0	0	0	0	1	0	0	0	0	0	0	0	0	0	0	0
PINO	0	2	23	3	0	8	1	3	15	6	0	1	0	2	27	9	27	258	9	7
PONI	0	0	0	0	0	0	0	0	0	0	0	0	0	0	0	0	0	0	0	1
PYOL	0	0	0	0	0	0	0	0	0	0	0	0	0	5	2	0	1	0	0	0
RHAT	229	3	0	0	4	0	95	1	0	15	8	5	0	7	1	30	0	0	0	0
RHCA	0	0	0	0	0	17	0	0	2	18	9	8	0	21	1	0	0	0	0	18
SAVI	0	0	0	0	0	0	0	0	0	0	0	0	0	1	0	0	0	0	0	0
SEAT	35	36	0	0	0	0	13	6	0	8	58	9	0	14	0	15	1	0	6	1
SECO	0	0	4	0	0	0	0	1	0	0	19	2	0	0	4	4	0	0	4	0
